# The Role of Active Site Residues in ATP Binding and Catalysis in the *Methanosarcina thermophila* Acetate Kinase

**DOI:** 10.3390/life5010861

**Published:** 2015-03-12

**Authors:** Cheryl Ingram-Smith, Jeffrey Wharton, Christian Reinholz, Tara Doucet, Rachel Hesler, Kerry Smith

**Affiliations:** Department of Genetics and Biochemistry, Clemson University, Clemson, SC 29634, USA; E-Mails: jwharto@g.clemson.edu (J.W.); rheino77@gmail.com (C.R.); tdoucet1@jhmi.edu (T.D.); rah33@duke.edu (R.H.)

**Keywords:** acetate kinase, acetate, ATP, *Methanosarcina*

## Abstract

Acetate kinase (ACK), which catalyzes the reversible phosphorylation of acetate by ATP, is a member of the acetate and sugar kinase/heat shock cognate/actin (ASKHA) superfamily. ASKHA family members share a common core fold that includes an ATPase domain with five structural motifs. The PHOSPHATE1 motif has previously been shown to be important for catalysis. We have investigated the role of two of these motifs in the *Methanosarcina thermophila* ACK (MtACK) and have shown that residues projecting into the ACK active site from the PHOSPHATE2 and ADENOSINE loops and a third highly conserved loop designated here as LOOP3 play key roles in nucleotide triphosphate (NTP) selection and utilization. Alteration of Asn211 of PHOSPHATE2, Gly239 of LOOP3, and Gly331 of ADENOSINE greatly reduced catalysis. In particular, Gly331, which is highly conserved throughout the ASKHA superfamily, has the greatest effect on substrate selection. Alteration at this site strongly skewed MtACK toward utilization of purines over pyrimidines, unlike the wild type enzyme that shows broad NTP utilization. Further investigation into differences between the ATPase domain in MtACK and other acetate kinases that show different substrate preferences will provide us with a better understanding of the diversity of phosphoryl donor selection in this enzyme family.

## 1. Introduction

Acetate kinase (ACK; EC 2.7.2.12), a member of the acetate and sugar kinase/heat shock cognate/actin (ASKHA) superfamily, catalyzes the reversible phosphorylation of acetate by ATP and is central to energy-yielding metabolism of anaerobes [1,2,3]. ACK functions with phosphotransacetylase (PTA; EC 2.3.1.8) to activate acetate to acetyl-CoA in the first step of the pathway for conversion of the methyl group to methane by *Methanosarcina* species from the *Archaea* [4]. During growth on carbon monoxide, the ACK-PTA pathway can also operate in the opposite direction for ATP production/synthesis [5,6].

Blattler and Knowles [7] showed that the reactions catalyzed by ACK, glycerol kinase, and hexokinase, all of which belong to the ASKHA superfamily, proceed with a net inversion of configuration of the transferred phosphoryl group indicating an odd number of phosphoryl transfers. Later studies with the *Methanosarcina thermophila* ACK (MtACK) showed that MgADP-AlF_3_-acetate forms a transition state analog in the active site and leads to an abortive complex [8]. A structure of ACK with this transition state analog revealed ADP-AlF_3_-acetate in a linear array in the active site [9], providing definitive support for the direct phosphoryl transfer mechanism. Based on analysis of site-altered enzyme variants and MtACK structural studies, Gorrell *et al.* [9] postulated a mechanism detailing the roles of active site residues in catalysis. The active site residues implicated in this mechanism are conserved among ACKs, consistent with their key roles in catalysis.

ASKHA family members have in common an ATPase domain with similar three-dimensional structure in which two subdomains form an active site cleft in which ATP at the bottom [1,2]. This ATPase domain consists of five sequence motifs, of which PHOSPHATE1, PHOSPHATE2, and ADENOSINE are involved in binding ATP and the CONNECT 1 and CONNECT 2 regions connect the two subdomains. The PHOSPHATE1 region is in one subdomain and the PHOSPHATE2 and ADENOSINE regions are in the other subdomain on the opposite side of the active site.

Several residues in the PHOSPHATE1 motif have been shown to be important for MtACK activity [10]. Crystal structures for MtACK show that residues in the PHOSPHATE1 loop are not within a distance for direct interaction with ATP [2,10]. However, ACK like other members of the ASKHA superfamily undergoes domain closure upon ligand binding [2,9,11] and this may bring the PHOSPHATE1 motif into close enough proximity to interact with the phosphate groups of ATP.

Here we investigated the roles of the conserved sequence motifs of the ATPase domain in nucleotide triphosphate (NTP) substrate selection and utilization. We focused on two highly conserved residues, Asn211 in PHOSPHATE2 and Gly331 in ADENOSINE, as these residues are very highly conserved in bacterial and archaeal ACKs but differ in either the fungal *Cryptococcus neoformans* ACK [12], which shows a strong preference for ATP over other NTPs, or the protist *Entamoeba histolytica* ACK, which utilizes PP_i_ instead of ATP [13]. We also examined Gly239, which projects into the active site as part of a highly conserved region, designated here as LOOP3, and hydrogen bonds with Asn211. Our results indicate that all three residues play a role in NTP selection and utilization, with Gly331 as a primary determinant.

## 2. Experimental Section

### 2.1. Materials

Chemicals were purchased from Fisher Scientific, VWR Scientific Products, Sigma Chemical or Gold Biotechnology. Oligonucleotides for site-directed mutagenesis were purchased from Integrated DNA Technologies.

### 2.2. Site-Directed Mutagenesis

Site-directed mutagenesis of the *M. thermophila ack* gene in pET*ack* [14] was performed using the QuikChange Site-Directed Mutagenesis Kit (Stratagene) according to the manufacturer’s instructions. Mutagenic primers were 33–36 nucleotides in length with the altered site(s) located in the middle. Mutations were confirmed by sequencing at the Clemson University Genomics Institute.

### 2.3. Heterologous Production and Purification of MtACK Enzymes

Cloning of the *M. thermophila ack* gene into pET15b has been previously described [14]. This construct provides for the addition of an N-terminal His_6_ tag for one-step affinity purification. The wild type and variant ACKs were heterologously produced in *Escherichia coli* BL21(DE3) (Novagen). Cells harboring the expression construct were grown at 37 °C shaking at 200 rpm until an A600 of ~0.8 was reached, and recombinant protein production was induced by the addition of IPTG to a final concentration of 0.5 mM. Cells were grown overnight at ambient temperature and harvested by centrifugation. Cell pellets were resuspended in buffer A (25 mM Tris, 150 mM NaCl, 10% glycerol, 20 mM imidazole, pH 7.4) and disrupted by three passages through a French pressure cell at ~138 MPa. The cell lysate was clarified by ultracentrifugation (2 h, 100,000× *g*). The supernatant was applied to a 5 mL HisTrap HP column (GE Healthcare) that had been equilibrated with buffer A. After extensive washing to remove unbound protein, the column was developed with a linear gradient from 20 mM to 500 mM imidazole. The protein peak was collected, dialyzed overnight in 25 mM Tris, 10% glycerol, pH 7.0, aliquoted, and stored at −20 °C. Protein concentration was determined by the Bradford method [15] using Bio-Rad Protein Assay Dye Reagent. The purity of each enzyme was determined by SDS-PAGE.

### 2.4. Enzymatic Assay for ACK Activity

Enzymatic activity was determined using the hydroxamate assay [16,17,18], which detects acetyl phosphate production by conversion to acetyl hydroxamate and subsequently to a ferric hydroxamate complex that can be detected spectrophotometrically at 540 nm. Although 65 °C is the optimal temperature for the enzyme [19], other studies on this enzyme have typically been performed at 37 °C [9,11,14,20,21,22], so all assays in this study were performed at that temperature for consistency. Assays were performed in 0.3 mL volumes in a reaction mixture containing 50 mM Tris (pH 7.5) and 300 mM hydroxylamine-HCl (pH 7.0) with the MgCl_2_:ATP and acetate concentrations varied. Reactions were initiated by the addition of enzyme and terminated by the addition of 1 volume of 10% trichloroacetic acid and 1 volume of 2.5% FeCl_3_ in 2 N HCl. The absorbance at 540 nm was compared against a standard curve prepared with acetyl phosphate to determine the amount of product formed. Reactions were performed in triplicate.

Assay optimization was performed iteratively to determine the final substrate concentrations to be used for each enzyme variant in determination of apparent kinetic parameters. Apparent kinetic parameters were determined by varying the concentration of one substrate while keeping the other constant at the optimal concentration. Nonlinear regression was used to fit the data to the Michaelis-Menten equation using KaleidaGraph (Synergy Software). Data shown represent the mean ± standard deviation.

## 3. Results and Discussion

### 3.1. The PHOSPHATE2, ADENOSINE, and LOOP3 Regions of Methanosarcina ACK Are Highly Conserved

Aceti and Ferry have previously shown that *Methanosarcina thermophila* ACK (MtACK) has a broad NTP substrate range, with the highest activity observed with ATP and TTP, and substantial activity with ITP, UTP, GTP, and CTP [16]. Likewise, the *Salmonella typhimurium* ACK (StACK) also has a broad NTP substrate range [23], but this is not the case for all ACKs. The *Cryptococcus neoformans* ACK (CnACK) shows a strong preference for ATP [12] (C. Ingram-Smith, T. Dang, A. Guggisberg, S. Henry, J. Welch, A. Mattison, and K. Smith, manuscript in preparation) whereas the *Entamoeba histolytica* ACK (EhACK) is unable to utilize NTPs as a substrate and instead uses PP_i_/P_i_ as the phosphoryl donor/acceptor pair [13]. Both of these enzymes also show a strong preference for the acetate-forming direction of the reaction, unlike MtACK which has high activity in both directions of the reaction [12,13,20]. With a number of ACK structures now available, we can begin to elucidate the molecular basis for this substrate preference or lack thereof.

**Figure 1:**
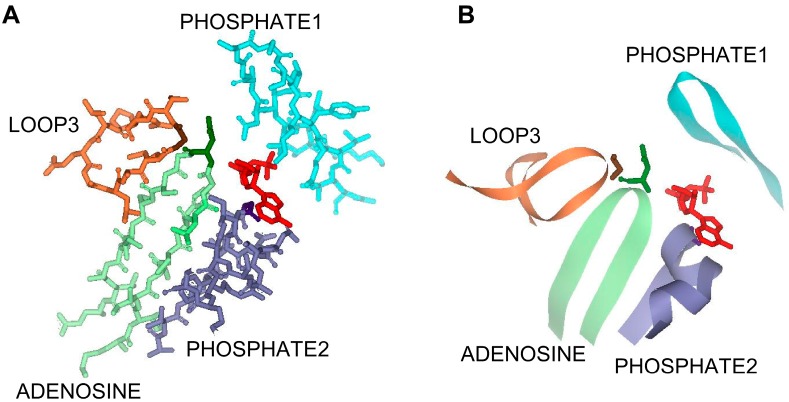
The active site region of the ATPase domain of MtACK (PDB: IG99) in (**A**) stick and (**B**) ribbon diagram. Residues shown conform to those in the alignment in Figure 2. ADP is shown in red, PHOSPHATE1 in light blue, PHOSPHATE2 is in light green with Asn211 in dark green, ADENOSINE is in light purple with Gly331 in dark purple, and LOOP3 is in light orange with Gly 239 shown in brown. For clarity, LOOP4 is not shown.

Three signature sequence motifs designated as ADENOSINE, PHOSPHATE1, and PHOSPHATE2 have been identified in the ASKHA superfamily of enzymes as playing a role in ATP binding [1,2,12,24]. In MtACK, the PHOSPHATE1 motif is located in the N-terminal domain on one side of the active site. The PHOSPHATE2 and ADENOSINE motifs are in the C-terminal domain located on the opposite side of the active site and each extends into the active site toward ATP (Figure 1). Of these, the PHOSPHATE1 motif has been studied the most in MtACK, with kinetic characterization of enzyme variants individually altered at five highly conserved residues within the motif [10]. The PHOSPHATE2 and ADENOSINE motifs have received less attention, with just two residues in each having been previously examined [14,20,22].

There are two additional loops, designated here as LOOP3 and LOOP4, that extend into the active site in this area (Figure 1). The residues in LOOP3 are highly conserved, whereas only two residues in LOOP4 are conserved. PHOSPHATE2, LOOP3, and ADENOSINE position Asn211, Gly239, and Gly331in close proximity to the ATP binding pocket. Arg285 of LOOP4 is located in the pocket that binds the adenosine moiety of ATP and lies planar to the adenine ring. Asn211 in PHOSPHATE2 interacts with the β-phosphate group, and also forms a hydrogen bond with Gly239. Gly331 in ADENOSINE interacts with both the α- and β-phosphate groups and is completely conserved among ACKs with the exception of the *E. histolytica* enzyme, suggesting it may play a key role in determining whether the enzyme utilizes ATP or PP_i_.

**Figure 2:**
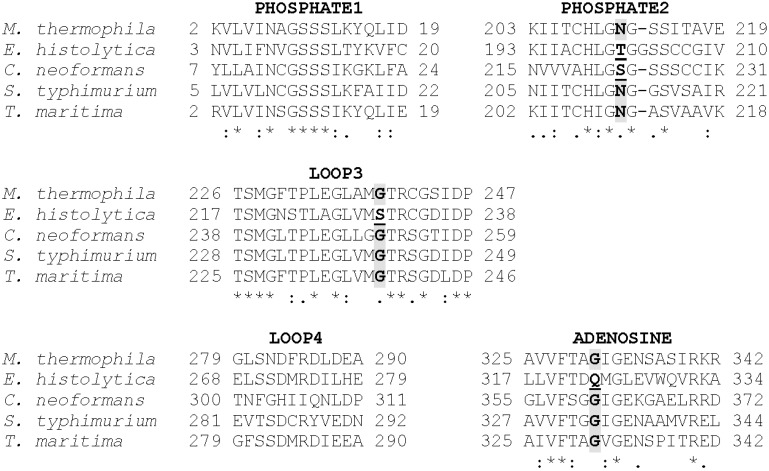
ACK sequence alignment showing the PHOSPHATE1, PHOSPHATE2, and ADENOSINE motifs as well as LOOP3 and LOOP4 which extend in toward the active site, as shown in Figure 1. Accession numbers: *M. thermophila*, GI:12084262; *E. histolytica*, GI:67481281; *C. neoformans*, GI:427930974; *S. typhimurium*, GI:16765664; *Thermotoga maritima*, GI:15643044.

Alignment of the MtACK sequence with the sequences from bacterial and eukaryotic ACKs whose structures are known shows high sequence conservation within the three signature motifs (Figure 2) as well as LOOP3 containing Gly239. Residues within LOOP4 are not well conserved with the exception of Arg285 and Asp283 with which it interacts. Notably, the four residues that project into the active site from the four regions shown are generally well conserved but are altered in either EhACK or CnACK, both of which have altered NTP utilization [12,13] (C. Ingram-Smith, T. Dang, A. Guggisberg, S. Henry, J. Welch, A. Mattison, and K. Smith, manuscript in preparation).

### 3.2. Asn211, Gly239, and Gly331 Influence NTP Preference and Utilization

In this study, we targeted Asn211, Gly239, and Gly331 for alteration to ascertain whether any of these residues influence NTP preference and utilization. In order to investigate the contribution of interactions between the enzyme and ATP to enzymatic activity, each of these residues was individually altered to Ala to remove side chain functionality, and to the alternative residues present in EhACK or CnACK. Asn211 in PHOSPHATE2 was replaced by Thr and Ser, the residues present in EhACK and CnACK, respectively. Gly331 in ADENOSINE and Gly239 in LOOP3 were replaced by Gln and Ser, respectively, to mimic EhACK. The variants were produced in *E. coli*, purified, and kinetically characterized.

The Asn211, Gly239, and Gly331 variants all had reduced specific activity *versus* the wild type enzyme, but to differing degrees (Table 1). The Asn211Ala, Gly331Ala, and Gly239Ala changes had the least effect, reducing activity by 6 to 15-fold. The Asn211Thr variant was moderately affected, with a 44-fold decrease in activity, and the Gly331Gln, Gly239Ser, and Asn211Thr alterations were the most deleterious, resulting in 117 to 200-fold decreased activity.

We examined NTP utilization by wild type MtACK and each variant. The wild type enzyme showed broad NTP utilization, with greater than 50% activity with CTP, GTP, TTP, UTP, and ITP *versus* ATP (Table 1). The Gly331 alterations had the most extreme effect. Activity of both Gly331 variants with the pyrimidine nucleotides CTP, TTP, and UTP was significantly reduced, with each displaying less than 12% activity *versus* ATP (Table 1). Activity with GTP and ITP was also reduced, but to a much lesser extent. Thus, it appears that the Gly331 alterations changed the substrate preference of the enzyme such that pyrimidines are preferred over purines, with ATP as the favored substrate.

**Table 1:** Activity of MtACK wild type and variants with each nucleotide triphosphate (NTP).

Enzyme	Specific Activity ^a^ (mol·min^−1^·mg^−1^)	Percent Activity Relative to That Observed with 10 mM ATP ^b^					
		ATP	CTP	GTP	TTP	UTP	ITP
Wild type	883 ± 13	100.0 ± 1.4	50.0 ± 0.4	85.2 ± 1.9	64.5 ± 0.7	54.8 ± 0.5	80.0 ± 0.5
Asn211Ala	145 ± 1.6	100.0 ± 1.1	56.4 ± 0.6	65.5 ± 1.1	78.6 ± 2.5	43.0 ± 0.3	80.0 ± 1.1
Asn211Ser	4.4 ± 0.04	100.0 ± 1.0	56.9 ± 0.6	65.7 ± 0.5	73.9 ± 0.4	43.7 ± 0.2	83.8 ± 0.7
Asn211Thr	20 ± 0.1	100.0 ± 0.6	55.7 ± 0.2	62.1 ± 0.9	95.7 ± 0.5	28.4 ± 0.3	75.4 ± 1.4
Gly239Ala	61 ± 1.7	100.0 ± 2.8	90.9 ± 1.0	64.3 ± 0.6	142.0 ± 0.9	60.2 ± 1.5	51.8 ± 0.5
Gly239Ser	4.6 ± 0.06	100.0 ± 1.2	93.7 ± 1.0	72.1 ± 0.7	163.4 ± 1.6	72.8 ± 0.7	56.2 ± 0.4
Gly331Ala	58 ± 1.0	100.0 ± 1.6	9.7 ± 0.5	49.0 ± 0.4	8.0 ± 0.3	4.4 ± 0.3	62.5 ± 0.1
Gly331Gln	7.5 ± 0.05	100.0 ± 0.6	11.8 ± 0.9	46.6 ± 0.5	8.1 ± 0.6	5.1 ± 0.4	69.0 ± 0.5

Notes: ^a^ Specific activity determined with 10 mM ATP; ^b^ Activities were determined at 10 mM final NTP concentration.

The Gly239 variants showed a shift in NTP utilization. Both variants displayed substantially higher activity with TTP than with ATP (Table 1), and activity (as a percentage of that observed with ATP) increased greatly with CTP as well. Only a weak increase in activity was observed with UTP. Activity with the purines GTP and ITP *versus* ATP decreased for both variants. Overall, the Asn211 variants had the least effect on NTP utilization. The Asn211Ala and Asn211Ser variants behaved similarly in terms of percentage activity observed with each NTP (Table 1). The percentage activity observed with CTP and ITP *versus* ATP was similar to that observed with the wild type enzyme. Activity with GTP and UTP decreased somewhat, and activity with TTP showed an increase. As for the Asn211Ala and Asn211Ser variants, the Asn211Thr variant showed little change in percentage activity observed with CTP and ITP, and the reduction in activity observed with GTP was similar to the other Asn211 variants. However, percentage activity observed with TTP and UTP was more substantially affected in the Asn211Thr variant. Activity with TTP was greatly enhanced and nearly equal to that observed with ATP, whereas the reduction in activity with UTP was stronger than that observed with the Asn211Ala and Asn211Thr variants.

We determined kinetic parameters for the wild type and each enzyme variant and found that the *K_m_* for ATP decreased slightly over two-fold for the Gly239 variants, but increased for all of the other variants (Table 2). The Asn211 variants had less than two-fold increased *K_m_* for ATP, but the Gly331 variants showed higher increases in the *K_m_* for ATP of 2.5-fold and 4.6-fold for the Ala and Gln variants, respectively.

**Table 2:** Apparent kinetic parameters for MtACK wild type and variants.

Enzyme	NTP	*K_m_* (mM)	*k*_cat_ (s^−1^)	*k*_cat_/*K_m_* (s^−1^·mM^−1^)	*K_m_* Acetate ^a^ (mM)
WT	ATP	4.0 ± 0.3	715 ± 10	180 ± 9	14.3 ± 0.5
-	CTP	3.2 ± 0.4	460 ± 28	148 ± 13	-
-	GTP	7.2 ± 0.9	571 ± 22	80 ± 7	-
-	TTP	2.7 ± 0.1	540 ± 9	202 ± 2	-
-	UTP	2.7 ± 0.3	415 ± 19	152 ± 7	-
-	ITP	4.7 ± 0.2	742 ± 10	158 ± 5	-
Gly331Ala	ATP	10.2 ± 0.4	88 ± 1.8	8.9 ± 0.18	601 ± 8
-	CTP	15.7 ± 1.6	17 ± 0.8	1.1 ± 0.07	-
-	GTP	5.3 ± 0.2	67 ± 1.0	12.6 ± 0.40	-
-	TTP	11.2 ± 0.5	20 ± 0.5	1.8 ± 0.05	-
-	UTP	*	43 ± 8.1	ND	-
-	ITP	10.7 ± 0.2	84 ± 0.4	7.8 ± 0.17	-
Gly331Gln	ATP	18.4 ± 0.6	16 ± 0.2	0.9 ± 0.02	**
-	CTP	11.1 ± 0.7	2.1 ± 0.05	0.2 ± 0.01	-
-	GTP	7.4 ± 0.4	8.6 ± 0.15	1.2 ± 0.04	-
-	TTP	12.1 ± 1.0	2.8 ± 0.10	0.2 ± 0.01	-
-	UTP	14.2 ± 0.5	2.8 ± 0.03	0.2 ± 0.01	-
-	ITP	10.1 ± 0.3	11 ± 0.1	1.0 ± 0.02	-
Asn211Ala	ATP	5.9 ± 0.5	374 ± 13	63.0 ± 5.7	165 ± 20
Asn211Ser	ATP	6.8 ± 0.6	13 ± 0.8	1.9 ± 0.1	140 ± 5
Asn211Thr	ATP	7.1 ± 0.3	32 ± 0.7	4.5 ± 0.2	470 ± 40
Gly239Ala	ATP	1.7 ± 0.1	77 ± 2.1	45.7 ± 2.6	113 ± 13.4
Gly239Ser	ATP	1.8 ± 0.1	6.8 ± 0.05	3.9 ± 0.19	94 ± 3.0

Notes: ^a^ Kinetic parameters for NTPs were determined at saturating acetate concentration when possible. The maximum acetate concentration used was 1 M. * Enzyme was not saturable for UTP. Kinetic parameters were determined with 25 mM UTP. ** Enzyme was not saturable for acetate.

We also determined *K_m_* for the other NTPs for the wild type enzyme and the Gly331 variants since they had the most extreme changes in NTP usage. For the wild type enzyme, *K_m_* values varied less than two-fold (from 2.7 mM to 4.7 mM), with the exception of GTP for which the *K_m_* value was 7.2 mM (Table 2). Slightly lower *K_m_* values were observed with pyrimidine nucleotides than purine NTPs. Overall though, the catalytic efficiencies *k*_cat_/*K_m_* do not indicate any specific preference for purine *versus* pyrimidine NTPs.

For the Gly331Ala and Gly331Gln variants, the *K_m_* values for NTP substrates showed increases of 2-fold to 13-fold *versus* the wild type enzyme with the exception of GTP (Table 2). The *K_m_* value for GTP decreased slightly for the Gly331Ala variant and was unchanged for the Gly331Gln variant. The *k*_cat_ values for the Gly331Ala variant decreased 8- to 26-fold, but there was no correlation between the level of reduction and whether the substrate was a purine or pyrimidine nucleotide. However, for the Gly331Gln variant the most severe reductions in *k*_cat_ were observed with pyrimidine nucleotides (148-fold to 219-fold decrease) *versus* the purine nucleotides (45- to 70-fold decrease). Overall, both the Gly331Ala and Gly331Gln variants displayed a preference for purine nucleotides over pyrimidine nucleotides as judged by the catalytic efficiency *k*_cat_/*K_m_*, unlike the wild type enzyme which did not show a distinct substrate preference.

MtACK Arg285Ala, Arg285Leu, and Arg285Lys variants have previously been analyzed and shown to have 6.8 to 8.6-fold reduced *k*_cat_ *versus* wild type, with 3 to 6-fold increased *K_m_* for ATP and ~6.8 to 11.5-fold increased *K_m_* for acetate [22], suggesting this residue is important for catalysis and substrate binding but is not essential. Given the overall lack of sequence conservation in LOOP4 and the planarity of Arg285 to the adenosine ring, we did not examine this residue further to determine if it plays any role in NTP selection.

ACK, like other members of the ASKHA enzyme superfamily, is proposed to undergo domain movement which would result in closure of the active site to sequester the substrates [2,3,11]. Of the four motifs identified in Figure 1, PHOSPHATE2, LOOP3, and ADENOSINE all reside in domain II, and only PHOSPHATE1 resides in domain I. Our results indicate that Gly331 in ADENOSINE may be responsible in large part for the broad NTP preference in MtACK. The other three motifs may play less of a role in determining substrate specificity but still play key roles in proper substrate positioning in the active site. The severe reduction in catalysis for the variants is consistent with this observation. Suboptimal substrate positioning may prevent proper domain closure, resulting in a catalytically less effective active site.

### 3.3. Substrate Specificity in MtACK versus Other ACKs

Yoshioka *et al.* [25] recently examined several residues in the ADENOSINE motif of *E. coli* ACK for their influence on ATP binding and ATP *versus* PP_i_ specificity. Gly332, Gly333, Ile334, and Asn337 (equivalent to Ala330, Gly331, Ile332, and Asn335 of MtACK) were altered to the residue present in EhACK. The *k*_cat_ was greatly reduced for each of these variants with the exception of the Asn337Glu variant, which had only ~2-fold reduced catalytic rate. All of the variants displayed an increased *K_m_* for ATP, particularly the Asn337Glu variant for which the *K_m_* increased over 45-fold. Yoshioka *et al.* interpreted these results to mean that Asn337 is the most crucial residue for specificity of ACK for ATP *versus* PP_i_ [25]. However, the relatively minor reduction in catalysis would argue against this. Their results are fully consistent with ADENOSINE playing a key role in ATP binding though.

In the crystal structures of MtACK [2,9,12], CnACK [12], and StACK [23], the nucleotide base binds in a hydrophobic pocket and does not make specific interactions with the enzyme. That alterations at Gly331 increased the preference for purines over pyrimidines suggests that addition of a side chain at this position may allow direct or indirect contacts between ADENOSINE and the six-membered ring of purines that are not possible for pyrimidine bases. However, the decreased activity of these variants indicates that although the purine base may be better accommodated, the active site architecture is no longer optimal for proper binding and orientation of both substrates for catalysis.

It is interesting to note that MtACK and StACK work well in both directions of the reaction and display broad NTP substrate specificity ([16,23] and this work), whereas CnACK and EhACK strongly prefer the acetate-forming direction of the reaction and have narrow substrate specificity. The broad *versus* narrow substrate specificity may reflect the physiological role of ACK in each microbe, serving to provide tighter control over the directionality of the reaction. In *Salmonella* and many other bacteria, the direction in which the ACK-PTA pathway functions depends on the cell’s need for ATP for metabolic processes *versus* acetyl-CoA as a building block [26]. In *Methanosarcina*, this pathway is used for the activation of acetate as a growth and energy substrate for methanogenesis, but can also operate in the reverse direction for energy conservation during growth under non-methanogenic conditions on carbon monoxide [5,6]. In *Cryptococcus* and other fungi, ACK partners with xylulose 5-phosphate/fructose 6-phosphate phosphoketolase (XFP) [27,28] in a modified pentose phosphoketolase pathway similar to that used in lactic acid bacteria in the heterofermentative degradation of pentoses and hexoses. In *E. histolytica*, the partner enzyme for ACK is unknown but ACK is proposed to provide PP_i_ for the unusual PP_i_-dependent glycolytic pathway [13].

## 4. Conclusions

Twelve conserved residues have previously been shown to be important for MtACK activity [9,10,11,14,20,22], and Ingram-Smith *et al.* [21] demonstrated that Val93, Leu122, and Pro232 are determinants for acetate binding in MtACK. These fifteen hallmark residues are highly conserved in both the prokaryotic and eukaryotic ACKs. Of these, Asn7, Ser10, Ser11, Ser12, and Lys14 belong to the PHOSPHATE1 motif.

We have identified three additional residues that are important for MtACK activity. Alterations at Asn211 and Gly239 in PHOSPHATE2 and LOOP3, respectively, reduced enzymatic activity but did not substantially influence NTP selection. Replacements at Gly331 in ADENOSINE not only reduced activity, but also changed the NTP preference of the enzyme. MtACK has a broad NTP range, with the highest activity observed with ATP followed by GTP and ITP, but with at least 50% activity with the pyrimidine NTPs. In scanning activity of the Gly331 variants with different NTPs, ATP was more strongly favored over the other purines and pyrimidine nucleotides were strongly disfavored. Our results suggest that although PHOSPHATE2 and LOOP3 may play a role in substrate positioning, the key role of the ADENOSINE motif may be in substrate specificity.
